# Sex differences in light sensitivity impact on brightness perception, vigilant attention and sleep in humans

**DOI:** 10.1038/s41598-017-13973-1

**Published:** 2017-10-27

**Authors:** Sarah L. Chellappa, Roland Steiner, Peter Oelhafen, Christian Cajochen

**Affiliations:** 1 Medical Chronobiology Program, Division of Sleep and Circadian Disorders, Departments of Medicine and Neurology, Brigham and Women’s Hospital, Boston, MA USA; 2 Division of Sleep Medicine, Department of Medicine, Harvard Medical School, Boston, MA USA; 3 Department of Physics, University of Basel, Basel, Switzerland; 4 Centre for Chronobiology, Psychiatric Hospital of the University of Basel, Transfaculty Research Platform Molecular and Cognitive Neurosciences, University of Basel, Basel, Switzerland

## Abstract

Artificial light endows a “round-the-clock”, 24-h/7-d society. Chronic exposure to light at night contributes to health hazards for humans, including disorders of sleep. Yet the influence of inter-individual traits, such as sex-differences, on light sensitivity remains to be established. Here we investigated potential sex-differences to evening light exposure of 40 lx at 6500 K (blue-enriched) or at 2500 K (non-blue-enriched), and their impact on brightness perception, vigilant attention and sleep physiology. In contrast to women, men had higher brightness perception and faster reaction times in a sustained attention task during blue-enriched light than non-blue-enriched. After blue-enriched light exposure, men had significantly higher all-night frontal NREM sleep slow-wave activity (SWA: 2–4 Hz), than women, particularly during the beginning of the sleep episode. Furthermore, brightness perception during blue-enriched light significantly predicted men’s improved sustained attention performance and increased frontal NREM SWA. Our data indicate that, in contrast to women, men show a stronger response to blue-enriched light in the late evening even at very low light levels (40lux), as indexed by increased vigilant attention and sleep EEG hallmarks. Collectively, the data indicate that sex differences in light sensitivity might play a key role for ensuring the success of individually-targeted light interventions.

## Introduction

The sharp delineation between day and night existing throughout most of our ancestral evolution has changed in industrialized society. A hallmark of industrialization is the increasing use of artificial light at night, which seems to coincide with a steady rise in a plethora of metabolic and oncogenic diseases^1^. In itself, this prompts the need to better understand how light at night impacts our physiology and behavior. In humans, exposure to light in the evening/night suppresses the release of the sleep-facilitating hormone melatonin^2,3^. Furthermore, it may shift the endogenous circadian clock to a later time^4^. These dual effects of light may result in a “dysfacilitation” to fall asleep at night. Apart from its impact on circadian physiology, light exposure acutely increases alertness^2,5–8^, cognitive brain function^9–11^, sleep onset^12^ and sleep EEG activity^13,14^. Indeed, we previously showed that blue-enriched light exposure attenuates frontal NREM sleep slow-wave activity^14^, a classical hallmark of sleep pressure. In other words, light enriched in the short-wavelength may acutely reduce sleep pressure. While long-term effects still remain to be established, a recent longitudinal 30-day study of daily smartphone measurements indicated that screen time was associated with shorter sleep duration and less sleep efficiency^15^. Inter-individual vulnerability to light may differentially impact on alertness, cognitive performance and sleep physiology. Indeed, specific genetic traits, including clock gene polymorphisms, have been ascribed to individual sensitivity to light at night that, in turn, may impact on subjective/objective alertness^16^, brightness perception and sleep EEG activity^17^. Thus, the impact of light on a wide-range of electrophysiological signatures may vary from person to person. Surprisingly, very little is known on how light is modulated by one of the most common traits of inter-individual differences: sex effects. Recently, circadian rhythmicity in cognitive function was shown to exhibit sex-differences, such that the nighttime impairment in cognitive performance is greater in women than in men^18^. However, it remains to be established if potential sex-differences in cognitive function and sleep regulation may also depend on how we respond to light exposure. Here we investigated whether evening light exposure to a higher (6500 K) and lower (2500 K) proportion of blue spectrum impacts on alertness, brightness perception, performance and sleep EEG activity, and if these effects are susceptible to a putative sex-related vulnerability.

## Results

### Light preference and subjective perception of brightness

**Figure 1:**
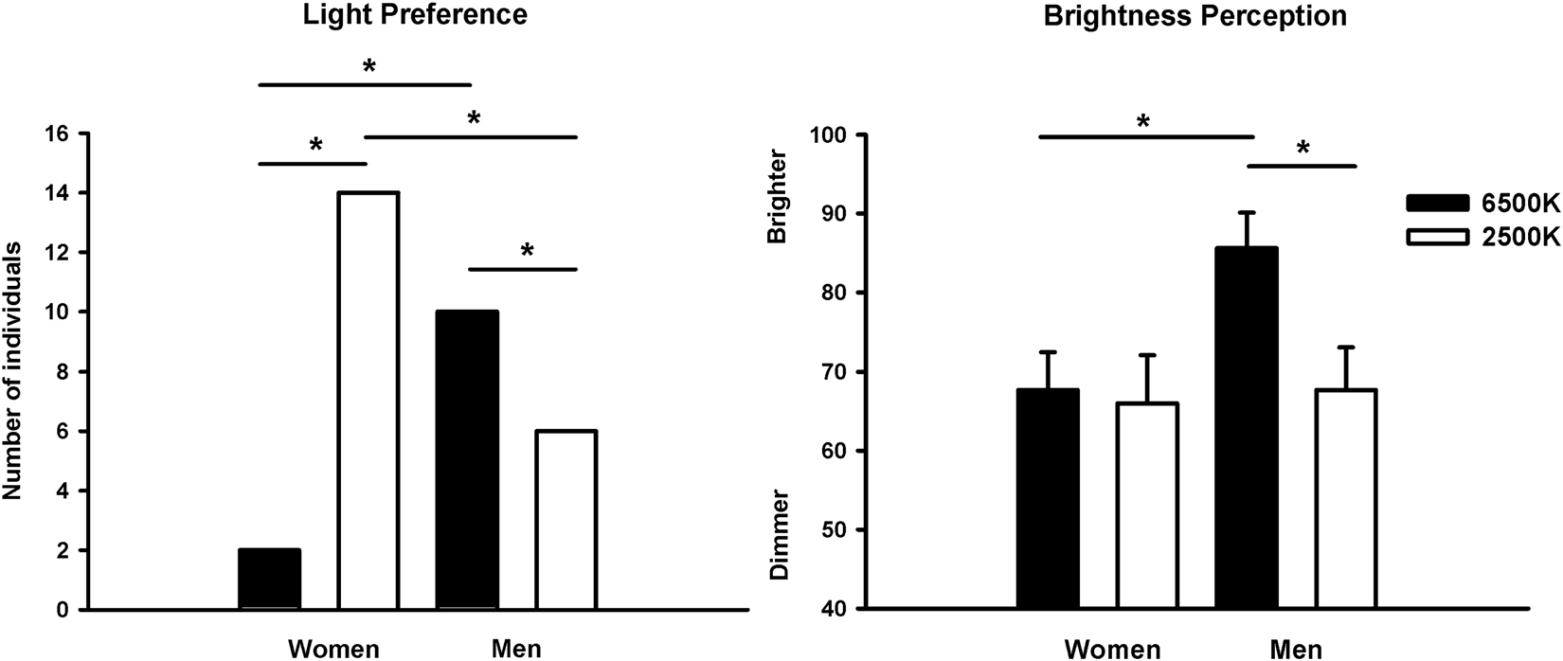
(**A**) Light color preference for women (*n* = 16) and men (*n* = 16) during exposure to light at 6500 K (black filled bars) and at 2500 K (black open bars). Data are presented as number of individuals. *Fisher’s Exact Test, *p* < 0.05. (**B**) Subjective perception of brightness as rated on a visual analogue scale from 0 to 100 for women (*n* = 16) and men (*n* = 16) during exposure to light at 6500 K (black filled bars) and at 2500 K (black open bars). Data are presented as mean ± standard error of mean. *Interaction of ‘light condition’ and ‘sex’, *p* < 0.05.

We observed a significant sex difference for light preference, such that men preferred light at 6500 K (62.5%) relative to 2500 K (37.5%), while the opposite was observed for women (6500 K: 12.5%, 2500 K: 87.5%) (Fisher’s Exact Test, *p* = 0.004) (Fig. 1A). Analyses of subjective perception of brightness yielded significant differences for main factors ‘light condition’ (*F*_1,29_ = 4.1; *p* = 0.04) and ‘sex’ (*F*_1, 29_ = 4.2; *p* = 0.04), and for the interaction of ‘light condition’ and ‘sex’ (*F*_1, 29_ = 3.9; *p* = 0.04) (Fig. 1B). Post-hoc analyses indicated than men perceived light at 6500 K as significantly brighter than at 2500 K (mean ± SEM: 85.6 ± 4.5 and 67.7 ± 5.4, respectively). Conversely, no significant differences were observed between light at 6500k and 2500 K for women (67.8 ± 4.8 and 66 ± 6.1, respectively).

### Sustained attention: Psychomotor Vigilance Task performance

**Figure 2:**
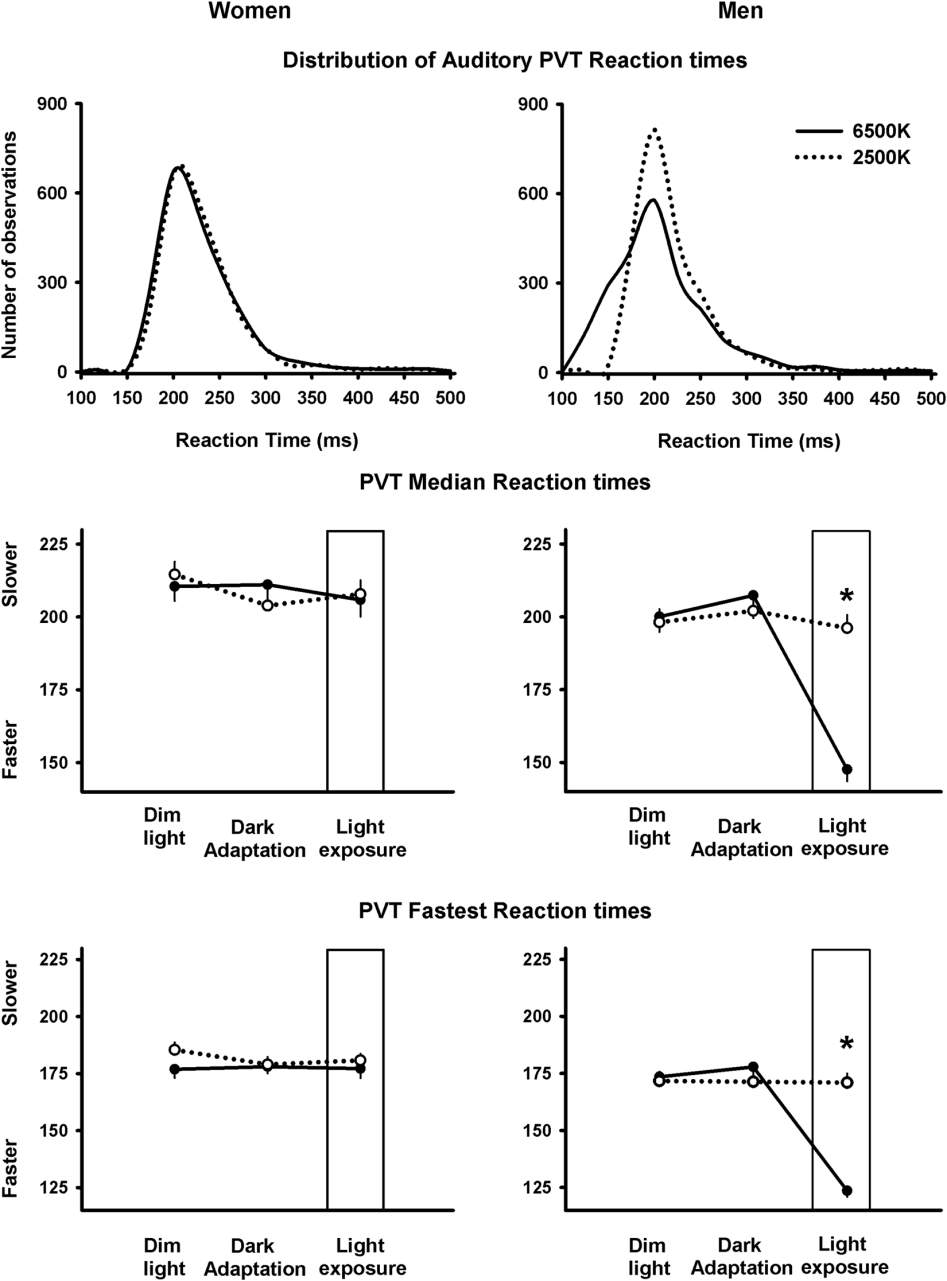
(**A**) Distribution of PVT reaction times for women (*n* = 16, left panel) and men (*n* = 16, right panel) during light exposure to 6500 K (black lines) and to 2500 K (dashed black lines lines). (**B**) Time-course of PVT median dim light (<8 lux), dark adaptation and reaction times during dim light and 2-h evening light exposure (white bar) at 6500 K (black lines) and at 2500 K (dashed black lines with open circles lines) for women (*n* = 16, left panel) and men (*n* = 16, right panel). (**C)** Same as for B, but for PVT performance was indexed as 10% fastest reaction times. Data illustrated as mean ± standard error of mean. *interaction of ‘light condition’, ‘session’ and ‘sex’, *p* < 0.05.

The distribution of PVT reaction times (number of observations of RT between 100–500 ms) indicated that light at 6500 K led to a shift towards the faster RT range as compared to light at 2500 K, *only* for men (Fig. 2A). Analyses of PVT performance revealed that main factor “light condition” elicited significance for median RT (F_1,56_ = 8.6, *p* < 0.005) and 10% fastest RT (F_1,59_ = 25, *p* < 0.001). Similarly, main factor “session” yielded significance for median RT (F_2,48_ = 29.2, *p* < 0.001) and 10% fastest RT (F_2,48_ = 29.4, *p* < 0.001). No main effects for “light condition” and “session” were observed for the slowest RT and for lapses (RT > 500 ms). Mixed-model analyses with the interaction of factors ‘light condition’, ‘session’ and ‘sex’ yielded significant differences for the median RT (F_4,48_ = 12.8, *p* < 0.001) and 10% fastest RT (F_4,48_ = 15.8, *p* < 0.001), such that light at 6500 K induced significantly faster reaction times compared to light at 2500 K, *only* for men (*p* < 0.05; Tukey–Kramer test) (Fig. 2B,C).

### Sleep structure and sleep EEG activity

**Figure 3:**
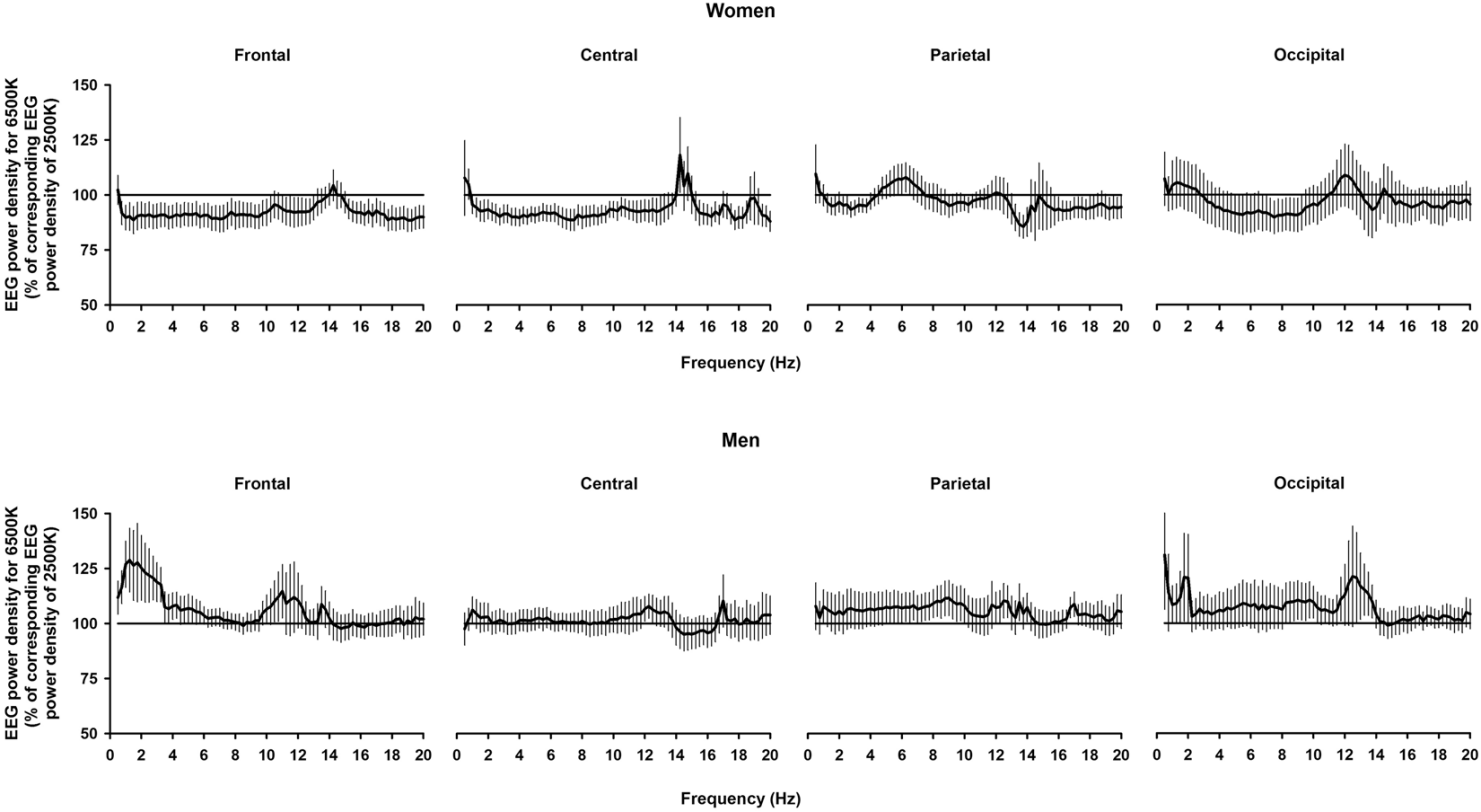
All-night EEG power density spectra during NREM sleep in frontal, central, parietal, and occipital derivations for women (*n* = 15, upper panels) and men (*n* = 16, bottom panels). NREM sleep EEG power density values per 0.25 Hz bins following light at 6500 K (black lines) are expressed as percentage of the corresponding average values following light at 2500 K (horizontal black line: 100% of EEG power density following light exposure at 2500 K). Data are presented as mean ± standard error of the mean of values for each 0.25 Hz frequency bin from 0.75 to 20 Hz. *Interaction of ‘light condition’ and ‘sex’; *p* < 0.05.

**Figure 4:**
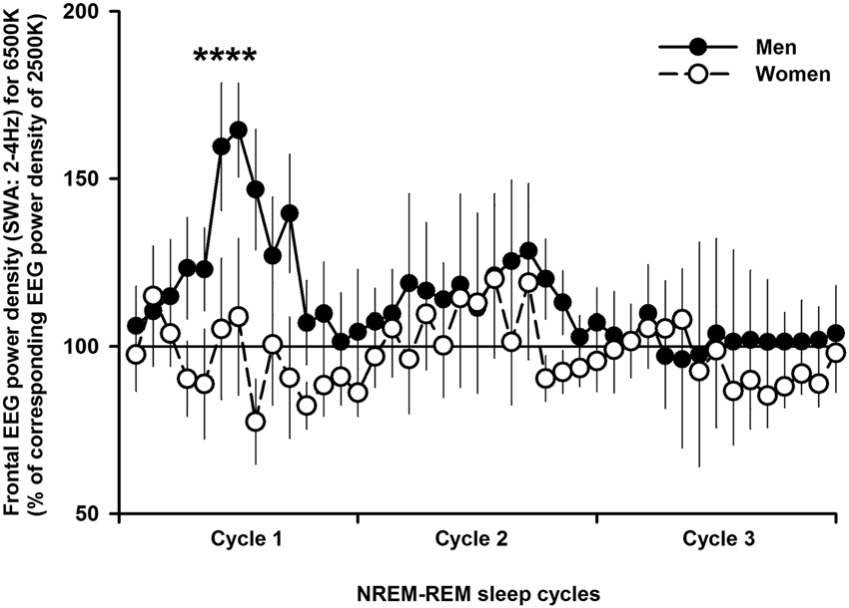
Dynamics of frontal EEG power density for NREM slow-wave activity (SWA: 2–4 Hz) during sleep cycles 1–3 for women (*n* = 15, open circle) and men (n = 16, black circle). NREM SWA EEG power density NREM-REM values are expressed as percentage of the corresponding average values following light sleep at 2500 K (horizontal black line: 100% of NREM SWA EEG power density values following light exposure at 2500 K). Data are depicted as mean ± standard error of mean. *Three-way r-ANOVA with ‘light condition’ vs. ‘sex’ vs. ‘percentiles’, *p* < 0.05.

On a next step, we investigated if inter-individual sensitivity to light impacts on sleep structure and sleep EEG activity. Mixed-model analyses with main factors ‘sex’ and ‘light condition’ and their interaction on all-night sleep structure yielded no significant sex differences for sleep structure between light conditions (data not shown). We then performed analyses on all-night NREM sleep EEG power density (absolute values for frequency bins in the range of 0.5–20 Hz). Mixed-model analyses with main factors ‘light condition’ and ‘sex’, for each derivation separately, yielded significant effects of ‘sex’ (*F*_1, 29_ > 5; *p* < 0.05) and of the interaction of ‘light condition’ and ‘sex’ (*F*_1, 29_ > 4; *p* < 0.05) for frontal NREM sleep SWA (range: 2–4 Hz). Figure 3 indicates that, subsequent to light at 6500 K, men had significantly higher frontal NREM SWA (2–4 Hz) sleep EEG power density as compared to women (*p* < 0.05; Tukey–Kramer test). All-night REM sleep EEG power density did not significantly differ between groups for both light conditions (data not shown). To investigate the dynamics of SWA (2–4 Hz) across sleep cycles, we analyzed SWA for each percentile during NREM-REM sleep cycles. Mixed-model analyses with main factors ‘light condition’, ‘sex’ and ‘percentiles’ performed separately for each derivation yielded significant effects of ‘sex’ (*F*_1, 206_ = 14.1; *p* = 0.002), ‘percentiles’ (*F*_41, 1136_ = 21.3; *p* < 0.001), and the interaction of ‘light condition’, ‘sex’ and ‘percentiles’ (*F*_123, 1975_ = 3.2; *p* = 0.03). Post-hoc comparisons yielded significantly more frontal NREM SWA during the first sleep cycle [time intervals 4–7 (percentiles) during NREM sleep)] following exposure to light at 6500 K, *only* in men (Fig. 4) (p < 0.05; Tukey–Kramer test). Individual data on the dynamics of frontal NREM SWA during the first NREM-REM sleep cycle further support these results, such that 11 out of 16 men (70%) had increased frontal NREM SWA, while this only occured in 5 out of 15 women (33%) (Supplementary Figure 1).

### Influence of subjective perception of brightness on PVT performance and NREM sleep SWA

**Figure 5:**
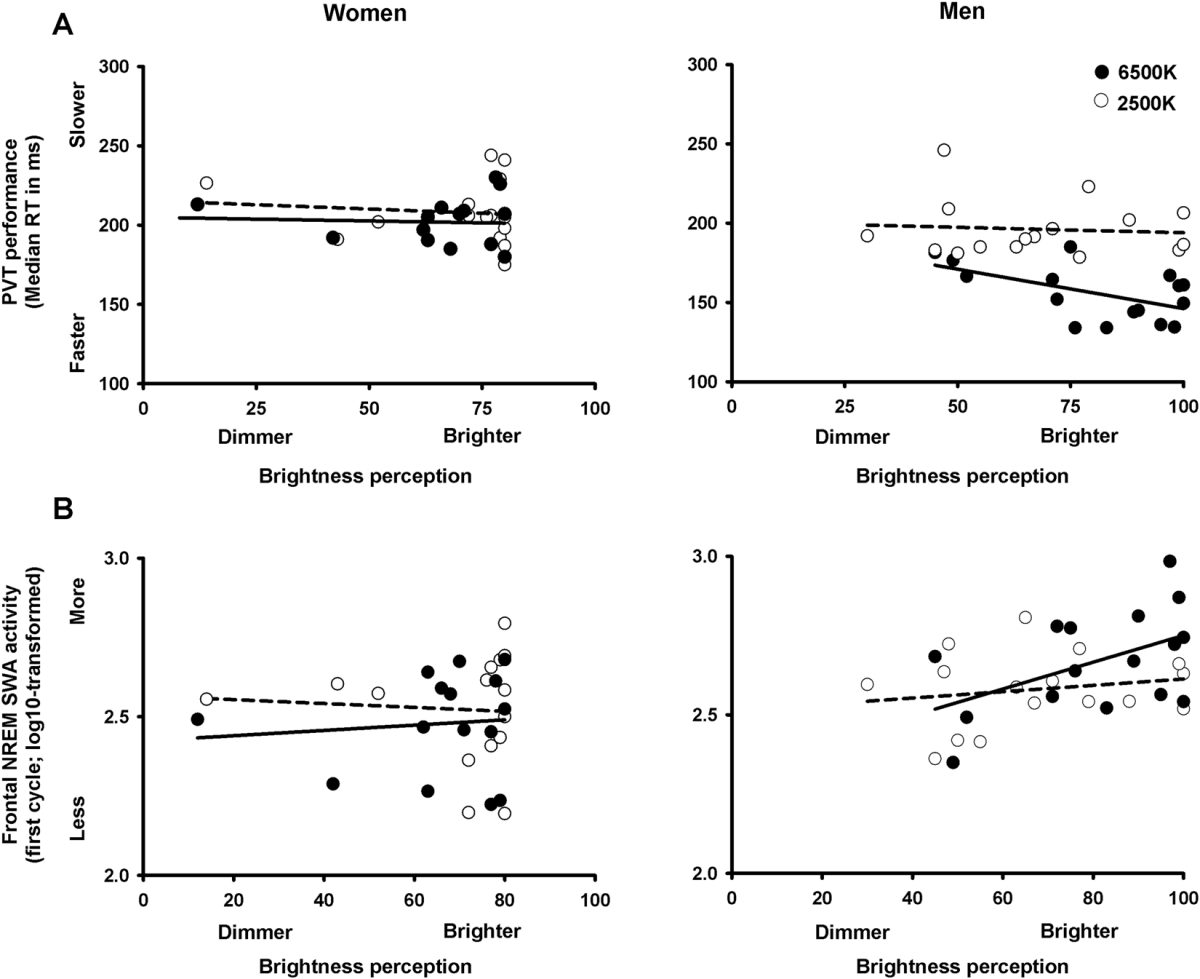
(**A**) Relationship between subjective perception of brightness (x-axis) and PVT performance (y-axis: median reaction times) for light exposure at 6500 K (black lines) and at 2500 K (dashed black lines with open circles) for women (*n* = 15, left panel) and men (*n* = 16, right panel). (**B**) Relationship between subjective perception of brightness (x-axis) and frontal NREM sleep slow-wave activity (y-axis: log10-transformed SWA, 2–4 Hz Hz) for light exposure at 6500 K (black lines) and at 2500 K (dashed black lines with open circles) for women (*n* = 15, left panel) and men (*n* = 16, right panel).

We then investigated whether subjective perception of brightness was associated to PVT performance, as indexed by median RTs. Analyses of covariance yielded significant differences for main factors ‘sex’ (*p* = 0.007), and ‘light condition’ (*p* = 0.004), and of the interaction of ‘sex’ and ‘light’ (*p* = 0.007). No significant relationship for brightness perception and PVT performance was elicited for light at 2500 K for both men and women. Conversely, these variables were significantly associated for light at 6500 K, *only* in men, such that the brighter they perceived light at 6500 K the faster were their reaction times **(**Fig. 5A **)**. On a next step, we investigated whether or not subjective perception of brightness was associated to frontal NREM sleep SWA (during the first NREM sleep cycle). Analyses of covariance yielded significant differences for main factors ‘sex’ (*p* = 0.04), of the interaction of ‘brightness’ and ‘sex’ (*p* = 0.008). No significant relationship for brightness perception and frontal NREM SWA was observed for light at 2500 K for both men and women. Conversley,  they were significantly associated for light at 6500 K, *only* in men, such that the brighter they perceived light at 6500 K the more frontal NREM sleep SWA they had in the subsequent sleep episode (Fig. 5B **)**.

## Discussion

We have evidence for a sex-dependent influence on subjective brightness perception, sustained attention and frontal NREM sleep EEG power density in the slow-wave activity range for blue-enriched light before bedtime. The magnitude of these effects depended on a putative inter-individual vulnerability to light, as there were only observed for men. Collectively, the results indicate that blue light in the hours before bedtime may acutely benefit sustained attention performance, and acutely impact on sleep physiology.

In humans, short-wavelength light in the evening/night induces higher circadian and alerting responses, as compared to light exposure to the same number of photons of longer-wavelength^2,12,19,20^, even though the shorter-wavelength light may have a much lower illuminance level as indexed by photopic lux^21^. Thus the seemingly detrimental impact on sleep may be ascribed to the artificial short-wavelength enriched light, which may elicit “carry-over” alerting effects onto the first part of the sleep episode^13,14^. Sleep induction by light may be modulated by melanopsin-based photoreception in animal models^22,23^, which speaks to a strong photic input to sleep. Direct photic input may alter the activity in the suprachiasmatic nucleus (SCN) and ventrolateral preoptic area (VLPO), the latter being implicated in sleep-wake regulation^24^. Melanopsin-based photic information might impinge onto the activity of these specific sleep-wake neural networks, therefore impacting sleep EEG activity.

Interestingly, we observed that subjective perception of brightness was associated with improved PVT performance and more frontal NREM SWA, possibly through distinct underlying mechanisms. Brightness is a shared construct between image and non-image forming systems^25,26^. This percept may associate with an object’s luminance, which comprises a photometric measure of light intensity. While it is relevant for cone photoreceptors, animal and human data also point to melanopsin involvement in brightness discrimination^25,26^. Thus, visually perceived brightness may be also encoded by melanopsin photoreception, impacting on non-image forming systems, as sleep physiology there after. Not only melanopsin but also other photopigments contribute to non-photic entrainment and responses^27–30^. Thus the association between brightness perception with both sustained attention and sleep EEG activity may be underlined by irradiance and color information.

Light at night may not impact the same way on everyone and our results speak to inter-individual sex-related vulnerability to light. The visual system function appears to be strongly influenced by sex differences^31,32^. Functional magnetic resonance imaging (fMRI) data on blood oxygen level dependent (BOLD) responses in primary visual cortex to stepped intensities of red and blue light indicate that stimulus-response curves for men and women were similar for red-light stimulus intensity^32^. Interestingly, for blue light, the stimulus-response curve in men (as indexed by the slope estimate curve) was twice as high than for women, suggesting a higher sensitivity to the blue spectrum of light. The underlying factors for a sex-related differential sensitivity to light remain to be fully established. Estrogen levels modulate dopamine activity, and pre-clinical studies suggest sex-specific variations in dopamine receptor density and dopamine turnover^33^. Dopamine is additionally involved in modulating retinal sensitivity and neurobiological responses to light, including light and dark adaptation in the retina^34^. It is tempting to speculate that putative sex differences in cortical responses to photic stimulation might be explained by variations in dopaminergic signaling.

We previously demonstrated that blue-enriched polychromatic light impacts on human sleep EEG activity, such that light exposure in the late evening may elicit a carry-over alerting effect on NREM EEG activity^14^. However, when splitting the data by sex, we observe that, while both men and women had similar frontal NREM slow-wave activity subsequent to light exposure at 2500 K, men had ~1.6 fold higher frontal NREM slow-wave activity after light exposure at 6000 K, as compared to women. Thus, it is likely that the previous finding on the blue-enriched alerting light effect on sleep EEG activity could be driven by the lower frontal NREM slow-wave activity in women. Here, we provide evidence that the effects of artificial light between dusk and sleep times on cognition and sleep are more nuanced than previously apprehended and may depend on sex-differences to light sensitivity. The potential mechanisms accounting for these sex-differences to light exposure are currently unknown. Future animal experimental work is needed to better understand the potential mechanisms by which sex differentially modulates light sensitivity and therefore sleep-wake regulation, which may occur at the retina level and/or signaling pathways from the retina to non-visual and visual brain areas.

Sex-differences to light exposure on cognition and sleep hold promising ramifications for individually targeted light exposure settings in real-life, including, but not limited to, vulnerable shift work populations. Yet, caution should be made when extrapolating our current findings. The impact of light is crucially relies on its specific properties, including intensity, duration, wavelength, dynamics and photic history^11^. To add complexity, time-of-day and the associated changes in the interaction between circadian and sleep homeostasis signals, as well as inter-individual trait markers as genetic polymorphisms, modulate the impact of light^14,16^. Thus, it is likely that potential sex-differences in light sensitivity may play a role on cognition and sleep in conjunction with a myriad of other key players. Importantly, we previously demonstrated that candidate gene polymorphims (*PER3* polymorphism) associated with sleep-wake regulation play a role in light sensitivity on sleep EEG activity^17^). In our current dataset, the genotype distribution of the *PER3* polymorphism in men is 7 *PER3 4/4*, 5 *PER3 4/5* and 4 *PER3 5/5* (total n = 16), and for women is 6 *PER3 4/4*, 8 *PER3 4/5* and 1 *PER3 5/5* (total n = 15). Thus, due to the low sample size per genotype group, we cannot disentangle potential interaction effects of “sex” and “genotype”. Therefore, to directly test and disentangle the effects of these two factors on light sensivity effects on sleep regulation, future studies with an appropriately powered study sampe would be required.

Electric light has endowed us with a 24-h/7-day society, to the extent that more than 90% of individuals in the US and Europe experience light at night^35^. It is not surprising that such all-around-the-clock exposure to light may foster multiple repercussions for physiology and behaviour. Indeed constant light exposure over 24-weeks in mice induces adverse effects on skeletal function, weight, glucose levels and a transient pro-inflammatory state, all of which reversible after reinstating standard light-dark cycles^36^. Chronic exposure to light at night may contribute to a plurality of health hazards for humans, including risk for breast cancer, heart disease, obesity and mood disorders^35^. Here we show that caution should be made when considering the acute effects of light exposure to the general population, such that inter-individual differences in light sensitivity should also be considered. Given the growing prevalence of light at night, attention is warranted to better understand how inter-individual vulnerability to light may underlie the physiological effects cascaded by this phenomenon. Ultimately, sex differences in light sensitivity may play a role for ensuring the success of individually-targeted light interventions in humans, including, but not limited to shift workers (i.e. industries, security and military personnel) where sustained attention and increased alertness is needed.

## Methods

### Participants

Detailed description of study participants, selection criteria and study protocol is provided elsewhere^8^. A total of 16 healthy men and 16 women (20–31 years; mean ± standard deviation: 25.2 ± 3.1, in years) were matched by age, body-mass index (BMI) and ethnicity. No significant differences were observed between groups for age, BMI and ethnicity (all Caucasians). The current study sample corresponds to the same as for^14^. In that study, data were collapsed across all participants irrespective of potential sex-differences, as it was unknown whether or not sex-differences could correspond to a trait marker for sleep-wake regulation. However, recent evidence speaks to potential sex-differences in the circadian rhythmicity of cognitive function; insofar the nighttime impairment in cognitive performance is greater in women than in men^18^. Here we investigated if our previous light exposure findings^14^ could be mediated by such inter-individual trait markers, which remain unknown to date. Furthermore, data from^14^ comprised 30 participants (16 men, 14 women). Sixteen women were enrolled for that study, out of which one was excluded from the EEG analyses due to poor EEG recordings (the same as for the current study) and one was excluded as this participant was prospectively enrolled based on genetic polymorhism traits. The rationale for this exclusion was that we only included participants whose genetic polymorhism traits were retrospectively assessed to ensure a random sample of population. Here we included the one women whose data were excluded from^14^ as the main outcomes of interest – brightness perception, vigilant attention and sleep EEG activity – did not differ from the other 14 women in this study sample (deviation was below 2x standard error of the mean for each variable of interest; single women vs. the mean of the other 14 women). This approach increased the study sample for women from 14 to 15 participants, thus ensuring a more balanced by-group comparison (16 men and 15 women).

All participants gave written informed consent. The study was approved by the local ethics committee (EKBB/Ethikkommission beider Basel, Switzerland) and conformed to the Declaration of Helsinki.

### Study design

A balanced cross-over design was carried out during the winter season (January to March), with three segments apart by 1-week. The protocol started nearly 10-h after each participants’ habitual wake-up time and ended the next day after usual wake-up time. Sleep–wake schedules were assessed by 1-week of wrist actigraphy (actiwatch L, Cambridge Neurotechnology Ltd., Cambridge, UK) and self-reported sleep logs. During each protocol, participants underwent 1.5-h under dim light (<8 lx), 2-h of darkness, 2-h of light exposure (compact fluorescent lamps with ca. 40 lx at 6500 K or at 2500 K or incandescent light bulbs with ca. 40 lx at 3000 K), and a post-light period of nearly 45-min under dim light (<8 lx) until habitual sleep time. Light at 40 lx was used since it is a typical indoor environmental intensity in naturalistic settings, during that time-of-day. Detailed information of light settings and study rationale are provided in^14^. Here we report data on light exposure to 6500 K (blue-enriched light) and 2500 K (non-blue-enriched light), as exposure to 2500 K and 3000 K had very similar effects.

### Light preference, subjective perception of brightness and cognitive performance

Light preference was indexed by asking participants which light condition they preferred the most at the very end of the study design (in the morning after all 3 light conditions were performed). To assess each participant’s subjective perception of visual comfort, we used a validated visual comfort scale^37^, which consists of a visual analogue scale with a 100 mm scale that probes brightness perception. Cognitive performance was indexed by a 5-min auditory psychomotor vigilance task (PVT), which was performed during dim light, dark adaptation and light exposure. PVT is a sustained attention performance task sensitive to circadian rhythmicity and sleep need^38,39^. Participants were requested to press the response button as fast as possible as soon they heard an auditory stimulus, which were presented in intervals randomly varying from 3 to 7s. All participants used the same laptop across the study in-lab segments, and the auditory tone was set at 60% of maximum threshold for all participants, which was defined based each participant’s auditory comfort level. Our primary auditory PVT outcome measures were median RT and for the 10% fastest reaction times, using a cut-off threshold of reaction times > 100 ms and <500 ms. Both PVT outcome measures and reaction time threshold are based on^8^.

### Polysomnographic recordings

Sleep EEG activity was continuously recorded during scheduled sleep with the Vitaport Ambulatory system (Vitaport-3 digital recorder TEMEC Instruments BV, Kerkrade, the Netherlands). Eight EEG derivations (F3, F4, C3, C4, P3, P4, O1, O2, referenced against linked mastoids, A1 and A2), two electrooculograms, two submental electromyograms, and two electrocardiograms were recorded. All signals were low pass filtered at 30 Hz (fourth order Bessel type anti-aliasing, total 24 dB/Oct) at a time constant of 1 s. After online digitization by using a 12 bit AD converter (0.15 μV/bit) and a sampling rate at 128 Hz for the EEG, the raw signals were stored on a flash RAM card (Viking, Rancho Santa Margarita, CA, USA) and later downloaded to a PC hard drive. Sleep stages were visually scored per 20-s epochs (Vitaport Paperless Sleep Scoring Software), according to^40^ by a single experienced polysomnography technician, blind to the light conditions. NREM sleep was defined as the sum of NREM stages 2, 3, and 4. Slow wave sleep (SWS) was defined as the sum of NREM sleep stages 3 and 4. EEG artifacts were detected by an automated artifact algorithm (CASA, 2000 PhyVision B.V., Gemert, Netherlands). Spectral analysis was conducted using a fast Fourier transformation (FFT; 10% cosine 4 s window), which yielded a 0.25 Hz bin resolution. EEG power spectra were calculated during NREM sleep and REM sleep in the frequency range from 0 to 32 Hz. Artifact-free 4 s epochs were averaged across 20 s epochs. Here we report EEG data for frontal (F3, F4), central (C3, C4), parietal (P3, P4) and occipital (O1, O2) derivations, in the frequency range of 0.75–20 Hz. Sleep recordings from 1 woman were excluded due to poor recordings.

### Statistical analysis

Statistical analyses were performed using SAS (version 9.1; SAS Institute, Cary, NC). Light preference between 16 men and 16 women was analyzed with a chi-square test. Subjective perception of brightness (16 men, 16 women) was assessed with mixed-model analyses of variance (PROC MIXED) with main factors ‘sex’ and ‘light condition’ (6500 K and 2500 K). For PVT performance, the well-established performance metrics median reaction time (RT), 10% fastest and 10% slowest RT and lapses were calculated according to^39^. Analysis of PVT performance (16 men, 16 women) was carried out with main factors ‘sex’, ‘light condition’ and ‘session’ (dim light, dark adaptation and light exposure) (PROC MIXED, SAS). LS means statement was used for post-hocs, and the Tukey–Kramer test was then used for the correction of multiple testing.

Visually scored sleep stages were expressed as percentages of total sleep time (TST). All-night EEG power density in NREM sleep (16 men, 15 women) was analyzed for frontal, central, parietal and occipital derivations for each 0.25 Hz frequency bin, with main factors ‘sex’, ‘light condition’ and ‘derivation’ (frontal, central, parietal, occipital). NREM-REM sleep cycles were defined according to^41^. Each sleep cycle was subdivided into 10 time intervals of equal length during NREM sleep and into four time intervals during REM sleep (percentiles). A mixed-model analysis of variance (PROC MIXED) was used with main factors ‘sex’, ‘light condition’, and ‘percentiles’, for each derivation separately (16 men, 15 women). All *p*-values were based on Kenward–Rogers corrected degrees of freedom (significance level: *p* < 0.05). LS means statement was used for post-hocs, and the Tukey–Kramer test was then used for correction of multiple testing.

Lastly, we investigated if inter-individual differences to light sensitivity impact on cognitive performance and sleep EEG activity. Thus, we tested the influence of subjective brightness perception during light exposure on 1) PVT performance (reciprocal transformation of median reaction times) and 2) NREM sleep SWA (log10-transformed SWA: 2–4 Hz, during the first NREM-REM sleep cycle). We used subjective brightness perception as a proxy for “light sensitivity”, which was entered in a covariate model (PROC GLM, SAS), in conjunction with main factors ‘brightness’, ‘sex’ and ‘light condition’, and their interactions on PVT performance and frontal NREM sleep SWA (Type III analyses to account for possible collinearity of main factors) (16 men, 15 women). Absolute values for brightness perception were used (Gaussian distribution), while a reciprocal transformation and log10 were, respectively, utilized for the median PVT reaction times and for NREM sleep SWA to meet parametric data distribution criteria.

## Electronic supplementary material

Supplementary material

## References

[1] Stevens RG, Brainard GC, Blask DE, Lockley SW, Motta ME (2014). Breast cancer and circadian disruption from electric lighting in the modern world. CA Cancer J Clin..

[2] Cajochen C (2011). Evening exposure to a light-emitting diodes (LED)-backlit computer screen affects circadian physiology and cognitive performance. J Appl Physiol..

[3] Chang AM, Aeschbach D, Duffy JF, Czeisler CA (2015). Evening use of light-emitting eReaders negatively affects sleep, circadian timing, and next-morning alertness. Proc Natl Acad Sci USA.

[4] Zeitzer JM, Dijk DJ, Kronauer R, Brown E, Czeisler CA (2000). Sensitivity of the human circadian pacemaker to nocturnal light: melatonin phase resetting and suppression. J Physiol..

[5] Cajochen C, Zeitzer JM, Czeisler CA, Dijk DJ (2000). Dose-response relationship for light intensity and ocular and electroencephalographic correlates of human alertness. Behav Brain Res..

[6] Cajochen C (2005). High sensitivity of human melatonin, alertness, thermoregulation, and heart rate to short wavelength light. J Clin Endocrinol Metab..

[7] Rahman SA (2014). Diurnal spectral sensitivity of the acute alerting effects of light. SLEEP..

[8] Chellappa SL (2011). Non-visual effects of light on melatonin, alertness and cognitive performance: can blue-enriched light keep us alert?. PLoS One..

[9] Vandewalle G (2006). Daytime light exposure dynamically enhances brain responses. Curr Biol..

[10] Vandewalle G (2007). Brain responses to violet, blue, and green monochromatic light exposures in humans: prominent role of blue light and the brainstem. PLoS One..

[11] Chellappa SL (2014). Photic memory for executive brain responses. Proc Natl Acad Sci USA.

[12] Santhi N (2012). The spectral composition of evening light and individual differences in the suppression of melatonin and delay of sleep in humans. J Pineal Res..

[13] Munch M (2006). Wavelength-dependent effects of evening light exposure on sleep architecture and sleep EEG power density in men. Am J Physiol Regul Integr Comp Physiol.

[14] Chellappa SL (2013). Acute exposure to evening blue-enriched light impacts on human sleep. J Sleep Res..

[15] Christensen MA (2016). Direct Measurements of Smartphone Screen-Time: Relationships with Demographics and Sleep. PLoS One..

[16] Chellappa SL (2012). Human melatonin and alerting response to blue-enriched light depend on a polymorphism in the clock gene PER3. J Clin Endocrinol Metab..

[17] Chellappa SL (2014). Light modulation of human sleep depends on a polymorphism in the clock gene Period3. Behav Brain Res..

[18] Santhi N (2016). Sex differences in the circadian regulation of sleep and waking cognition in humans. Proc Natl Acad Sci USA.

[19] Boyce PR (2004). Lighting research for interiors: the beginning of the end or the end end of the beginning. Lighting Res Technol..

[20] Lockley SW, Brainard GC, Czeisler CA (2003). High sensitivity of the human circadian melatonin rhythm to resetting by short wavelength light. J Clin Endocrinol Metab..

[21] Lockley SW (2006). Short-wavelength sensitivity for the direct effects of light on alertness, vigilance, and the waking electroencephalogram in humans. SLEEP..

[22] Lucas RJ (2014). Measuring and using light in the melanopsin age. Trends Neurosci..

[23] Tsai JW (2009). Melanopsin as a sleep modulator: circadian gating of the direct effects of light on sleep and altered sleep homeostasis in Opn4(−/−) mice. PLoS Biol..

[24] Lupi D, Oster H, Thompson S, Foster RG (2008). The acute light-induction of sleep is mediated by OPN4-based photoreception. Nat Neurosci..

[25] Aston-Jones G (2005). Brain structures and receptors involved in alertness. Sleep Med..

[26] Brown TM (2010). Melanopsin contributions to irradiance coding in the thalamo-cortical visual system. PLoS Biol..

[27] Brown TM (2012). Melanopsin-based brightness discrimination in mice and humans. Curr Biol..

[28] Lall GS (2010). Distinct contributions of rod, cone, and melanopsin photoreceptors to encoding irradiance. Neuron..

[29] Altimus CM (2010). Rod photoreceptors drive circadian photoentrainment across a wide range of light intensities. Nat Neurosci..

[30] Walmsley L (2015). Colour as a signal for entraining the mammalian circadian clock. PLoS Biol..

[31] van Diepen HC, Foster RG, Meijer JH (2015). A colourful clock. PLoS Biol..

[32] Hedera P (1998). Sex and electroencephalographic synchronization after photic stimulation predict signal changes in the visual cortex on functional MR images. AJNR Am J Neuroradiol..

[33] Cowan RL (2000). Sex differences in response to red and blue light in human primary visual cortex: a bold fMRI study. Psychiatry Res..

[34] Walker QD, Rooney MB, Wightman RM, Kuhn CM (2000). Dopamine release and uptake are greater in female than male rat striatum as measured by fast cyclic voltammetry. Neuroscience..

[35] Witkovsky P (2004). Dopamine and retinal function. Doc Ophthalmol..

[36] Navara KJ, Nelson RJ (2007). The dark side of light at night: physiological, epidemiological, and ecological consequences. J Pineal Res..

[37] Lucassen EA (2016). Environmental 24-hr Cycles Are Essential for Health. Curr Biol..

[38] Dinges DF (1997). Cumulative sleepiness, mood disturbance, and psychomotor vigilance performance decrements during a week of sleep restricted to 4-5 hours per night. SLEEP..

[39] Graw P, Krauchi K, Knoblauch V, Wirz-Justice A, Cajochen C (2004). Circadian and wake-dependent modulation of fastest and slowest reaction times during the psychomotor vigilance task. Physiol Behav..

[40] Rechtschaffen, A. & Kales, A. A manual of standardized terminology, techniques and scoring system for sleep stages of human subjects. US Dept of Health, Education and Welfare, Public Health Service, Bethesda, MD (1968).

[41] Feinberg I, Floyd TC (1979). Systematic trends across the night in human sleep cycles. Psychophysiology..

