# Supplementary material for: Sex differences in light sensitivity impact on brightness perception, vigilant attention and sleep in humans
Source: Sci Rep. 2017 Oct 27;7:14215. doi: 10.1038/s41598-017-13973-1 (PMC5660221; doi:10.1038/s41598-017-13973-1)
Supplement: Supplementary file 1 — Supplementary material [file 41598_2017_13973_MOESM1_ESM.pdf]

Sex differences in light sensitivity impact on brightness perception, vigilant attention and sleep in humans

Sarah L. Chellappa[1,2], Roland Steiner[3], Peter Oelhafen[3], Christian Cajochen[4]*

[1] Medical Chronobiology Program, Division of Sleep and Circadian Disorders, Departments of Medicine and Neurology, Brigham and Women's Hospital, Boston, MA, USA

[2] Division of Sleep Medicine, Department of Medicine, Harvard Medical School, Boston, MA, USA

[3] Department of Physics, University of Basel, Basel, Switzerland

[4] Centre for Chronobiology, Psychiatric Hospital of the University of Basel, Transfaculty Research Platform Molecular and Cognitive Neurosciences, University of Basel, Basel, Switzerland

Supplementary Figure and Supplementary Figure legend

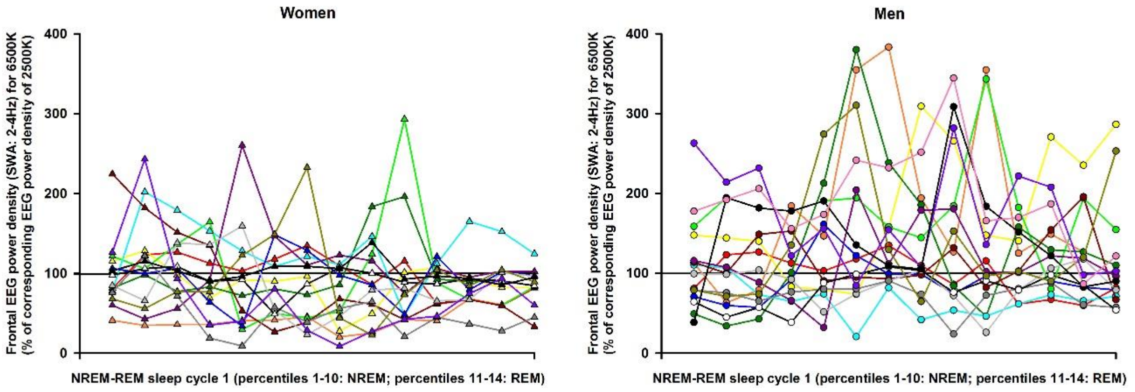

Supplementary Figure 1. Individual time-course of the dynamics of frontal NREM SWA (2-4Hz) during the first NREM-REM sleep cycle in women (left panel, *n*=15) and men (right panel, *n*=16). NREM SWA EEG power density values are expressed as percentage of the corresponding average values following light at 2500K (horizontal black line: 100% of NREM SWA EEG power density values following light exposure at 2500K). Each coloured line represents a participant.
